# Correlation Between Learning Motivation and Satisfaction in Synchronous On-the-Job Online Training in the Public Sector

**DOI:** 10.3389/fpsyg.2022.789252

**Published:** 2022-07-12

**Authors:** Nathan Cheng-Hu Chow, I-Jan Yeh

**Affiliations:** Department of Public Policy and Management, Shih Hsin University, Taipei City, Taiwan

**Keywords:** public sector, on-the-job training, synchronous online learning, learning motivation, satisfaction

## Abstract

Non-governmental organizations often regard expanding revenue and reducing costs as standard procedures to achieve corporate sustainability, while at the same time considering human resources as important assets. Government agencies have greater flexibility in staffing, and their human resource strategies for employee education and training often use organizational learning to develop operational performance. Training is regarded as a panacea for corporate sustainability and channels have been established to support employees' learning. Curriculum development of synchronous online learning is an approach that requires further investigation. We distributed 360 questionnaires to supervisors and employees of the Taipei City Government, Taiwan. A total of 268 valid copies were retrieved, giving a response rate of 74%. The study results are expected to help public sector employers enhance employee cohesiveness and generate more operational team spirit.

## Introduction

Non-governmental organizations often aim to achieve corporate sustainability by expanding revenue and reducing costs. Although human resources are regarded as an important company asset, at the same time, employees are asked to maximize the production capacity. Each employee has unique characteristics and business owners typically try to maximize individual performance for corporate sustainability. Government agencies—in contrast with non-governmental organizations—have greater flexibility in staffing. In the wake of globalization, information transparency, and global competitiveness, governments have prioritized the development of methods and strategies to promote competitiveness. To maintain a national competitive advantage and meet citizens' expectations, there is an urgent need to improve the skills of employees in public sector organizations.

Traditional models of education and training emphasize professional knowledge and technologies, and might involve a group of people sitting in a classroom listening to a lecturer—a passive “armchair” strategy. To broaden and deepen education, the Taiwanese government is dedicated to promoting digital learning activities for public servants through the application of information technology to digital learning. The aim is to improve the competence of the workforce and their professional skills. Many public sector agencies are aware of the importance of digital learning and are developing plans to promote digital learning and establish a public sector digital learning network with relevant courses for civil servants. Digital learning will be promoted and used to improve the professional skills of civil servants and promote the competitiveness of the government.

Organizational learning has become more popular in recent years. Training employees to advance operational performance is sometimes considered a panacea for corporate sustainability. Following this trend, the public sector has established many learning channels for employees, including curriculum development of synchronous online learning. Advances in information technology and the Internet have facilitated changes and growth across industries. In the context of dynamic business strategies and technologies, employees must grow and adapt to cope with the changeable working environment, especially when seeking promotion. Computers have led to widespread Internet use and changes in educational trends and the way people acquire knowledge. Mobile learning is a continuous learning approach that facilitates learning through action. Such active learning is more effective than traditional approaches that involve memorizing information, and advantages include longer-term impacts for organizations.

The methods and skills of private enterprises can be applied to research on human resources and the organizational characteristics of the public sector. Even though the recruitment, assessment, training, counseling, and retirement systems of government agencies are largely different from those of non-governmental organizations, these approaches could affect and benefit employee training. On-the-job training could directly enhance professional knowledge and skills and change individuals' professional attitudes. The training may also improve service quality, and indirectly and intangibly strengthen confidence in personal ability and job satisfaction. On-the-job training in the public sector could enable these organizations to cope with the rapidly changing environment. Organizations would be better able to meet professional standards and guarantee service quality. However, on-the-job training may need to be suspended in public sector workplaces when crises or accidents occur (Chen et al., 2020).

Bad weather, transportation challenges, and pandemics such as SARS and COVID-19 may create situations where large groups and in-person activities should be avoided. In non-pandemic scenarios, this might involve an interruption in knowledge delivery. However, the delivery and acquisition of information become more important in response to an epidemic (Yang et al., 2004). To overcome such situations, synchronous online learning, not restricted by time and space, could be used for on-the-job training (Richarson and Swan, 2003). Accordingly, we focus on the correlation between learning motivation and satisfaction with synchronous online on-the-job training in the public sector. The findings may contribute to the enhancement of employee cohesiveness to increase operational team spirit in public sector workplaces.

## Literature Review and Hypothesis

Sung and Hwang (2018) discuss the broad application of e-learning to on-the-job training in Europe and the US. In-service staff cannot always participate in regular classes because of their work commitments and multiple roles. The accessibility of e-learning removes the learning limitations for in-service staff without them having to leave home. Stouthuysen et al. (2018) used traditional teaching and e-learning to teach nursing to college students, and found no difference in learning outcomes. Students considered convenience, flexibility, and saving money as the main factors for choosing distance learning courses to enhance their learning motivation. In-service staff considered the flexible curricula of active learning and the provision of alternative routes for learning as factors that enhanced their learning motivation and enabled effective learning (Kamal et al., 2020). The following hypothesis is therefore proposed in this study:

**H1**: Synchronous online learning has positive and significant effects on learning motivation.

Gu et al. (2019) revealed that e-learning could enhance learning interests in terms of information ability and satisfaction. They suggested that hospitals and clinics should reinforce computer information courses and software/hardware facilities through which nursing staff can acquire learning resources. Meanwhile, hospitals and clinics should consider purchasing additional technology equipment and establishing certificate specifications for standard courses to enhance employees' learning motivation and effectiveness. Oliveira et al. (2018) revealed that all participants in their study were satisfied or extremely satisfied with learning, after participating in cooperative learning aided by an e-learning platform. They agreed that online learning within a cooperative community makes learning more effective for members. All members completed case reports and met the required standard, indicating effective skill learning using the e-learning platform. Hopp et al. (2018) applied synchronous e-learning using the Internet to enable nursing colleagues' learning. The practical value of e-learning for nursing education was shown and nursing staff reported relatively enhanced satisfaction. As a result, the present study evaluates the following hypothesis:

**H2**: Learning motivation has significant positive effects on learning satisfaction.

Ha and Nguyen (2019) studied the relationship between learning motivation and learning satisfaction and discovered that learners with stronger participation motivation were more satisfied with the learning activity. Statistical analyses indicated significant positive effects of learning motivation on learning satisfaction, i.e., students with stronger learning motivation rated their learning satisfaction more highly. Yahia et al. (2018) discovered that learners' motivation before participation could better predict their satisfaction with teachers than their learning performance and progress. High satisfaction with electronic and distance education was not necessarily because of high motivation, while low satisfaction was mostly linked to low motivation (Layona et al., 2018). The following hypothesis is further proposed in this study.

**H3**: Synchronous online learning has a positive effect on learning satisfaction.

## Methodology

**Figure 1:**
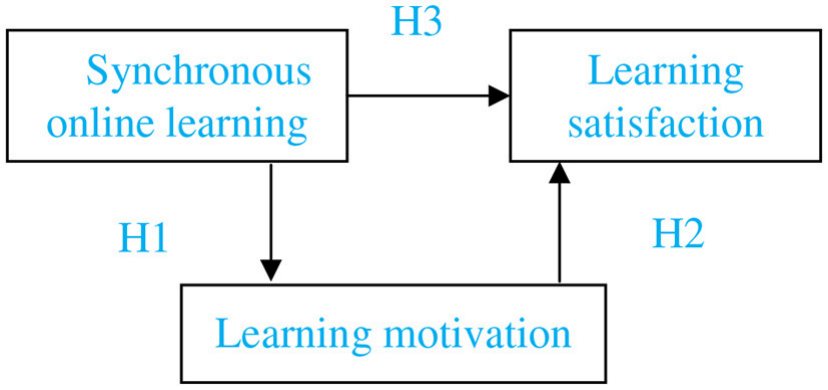
Conceptual structure.

The conceptual structure of this study is based on the literature review and is shown in Figure 1.

The contact persons for public sector units were contacted first to confirm that they have offered synchronous on-the-job online training learning before and were willing to distribute the questionnaire. To ensure that the sample composition was proportionate to the population, civil servants of all genders, seniority, grades, and titles were included and questionnaires were evenly distributed across these groups without concentrating on a single background or community variable. A written notice was then sent to the sampled public sector units together with the questionnaire.

### Operational Definitions

#### Synchronous Online Learning

Chen and Tsai (2019) describe a synchronous online learning model in terms of role (instructors, learners), participation method (individual, group), participation location (designated location, random locations), interactive mode (one way, two way), and course delivery method (playing course recorded content, real-time instruction, mixed method). The five dimensions are explained below together with the relevant items included in our questionnaire.

##### Role

Synchronous online learning comprises instructor and learner roles, with the sole requirement that teachers and students have access to the virtual classroom at the same time. Items included: (1) Learners have greater autonomy in the learning process than in traditional education, (2) Instructors individually address learner characteristics in distance learning, and (3) Distance learning materials are designed to meet learners' needs.

##### Participation Method

The participation method of synchronous online learning includes individual and group learning. Students may participate in cooperative learning or group discussions supported by individual learning. Items focusing on the participation method included: (1) Learners can develop full self-control in the learning process, (2) Distance learning is a learner-centered learning style, and (3) Learners can learn according to personal needs in the learning process.

##### Participation Location

Participation in synchronous online learning can occur anywhere or in a designated location. Teachers and students may participate in online teaching and learning at locations that provide personal and flexible learning spaces and an environment tailored to their needs. Items included: (1) Learning is not restricted to time, (2) Synchronous online learning can take place anywhere, and (3) Time can be flexibly used for synchronous online learning.

##### Interactive Mode

Interactive mode refers to learning and interaction between teachers and students and among students. The interactive mode for synchronous online learning could be a one-way transmission, e.g., real-time multicast, or two-way interaction, e.g., online real-time discussion. Items included: (1) Teacher–student interaction can be achieved through various methods, (2) Learning is not affected by classmates, and (3) Synchronous online learning can rapidly provide sufficient interaction information.

##### Delivery Method

Synchronous online learning courses can be delivered by teachers playing recorded files, real-time online teaching, or a mix of the two approaches. Items included: (1) I can learn to use synchronous online learning by observing others use it, (2) The materials provided in synchronous online learning meet the learning needs, and (3) The operation response time of synchronous online learning is fast.

#### Learning Motivation

Referring to Kim et al. (2019), online learning motivation included usefulness and enjoyment in this study. The two dimensions are explained below together with an outline of the organization of questionnaire items.

Usefulness, a form of extrinsic motivation, refers to individuals using online learning to provide data for work or help solve problems. Items included: (1) Career promotion, (2) Learning knowledge and skills related to work, (3) Recognizing the importance of continuous learning in the working process, (4) Being open to relevant education because of changes in work, and (5) Promoting personal professional image.

Enjoyment, an example of intrinsic motivation, refers to the use of online learning because of feelings of happiness and satisfaction. Items included: (1) Enjoying learning new knowledge and new concepts, (2) Interest in the course, (3) Broadening horizons and enriching personal knowledge, (4) Learning new knowledge to compensate for inadequacies, and (5) Continuous development because of changing knowledge.

#### Learning Satisfaction

Referring to Chien et al. (2019), course content, teaching methods, and learning methods were used as the dimensions for measuring learning satisfaction in this study. The three dimensions and their organization are explained below.

Course content refers to new course knowledge, focusing on helpful content, and rich and diverse courses. Items covered were: (1) It becomes natural to interact with learners or instructors through online teaching platforms, (2) The discussion in online learning is high quality, (3) It is easy to participate in discussions in online learning, (4) The interaction between instructors and learners in online learning is more difficult than in traditional teaching, and (5) I am satisfied with online learning.

Teaching method considers the evaluation methods used for assignments, multimedia-based teaching methods, teaching interactivity of courses, and lively teaching methods. Items included: (1) I am satisfied with online learning, (2) I would like to participate in online learning at any opportunity, (3) I think online learning is a smart decision, (4) I am satisfied with online learning, and (5) I think online learning meets my needs.

Learning method includes: the operation of interface, learning at home, learning time control, and repeatedly watching course content. Items included: (1) Compared with traditional teaching, I think the quality of online learning is better, (2) I think the quality of online learning is better than traditional teaching styles, (3) Compared with traditional teaching styles, learning becomes more difficult for me with online learning, (4) Compared with traditional teaching styles, the interaction among colleagues is easier in online learning, and (5) Compared with traditional teaching styles, participation in discussions is easier in online learning.

### Research Sample and Participants

Supervisors and employees of Taipei City Government, Taiwan were the participants in this study. We distributed 360 copies of the questionnaire, and 268 valid copies were returned, giving a retrieval rate of 74%. The public sector units and their contact persons were first selected to confirm that the units had had exposure to synchronous on-the-job online learning before and were willing to distribute the questionnaire. A written notice was then sent to the sampled public sector units with the questionnaire.

### Reliability and Validity

Confirmatory Factor Analysis (CFA) is an important part of Structural Equation Modeling (SEM). The measurement model should be tested before the two-stage model modification when using CFA for the structural model. When the model fit is acceptable, the second step of SEM can follow. In this study, the analysis of dimensions in CFA revealed factor loadings of 0.60–0.90, composite reliability of 0.70–0.90, and the average variance extracted (AVE) of 0.60–0.80, conforming to the standards of factor loading higher than 0.5; composite reliability higher than 0.6, and AVE higher than 0.5. The dimensions, therefore, indicate convergent validity.

## Results

### Sample Structure

The sample structure is summarized in Table 1.

**Table 1:** Sample structure.

**Demographic** **variable**	**Item**	**No. of** **participants**	**Percentage** **%**	**Total**
Grade	Supervisor	85	31.7	268
	Employee	183	68.3	
Gender	Male	153	57.1	268
	Female	115	42.9	
Age (years)	20 or younger	6	2.2	268
	20–30	74	27.6	
	31–40	103	38.4	
	41–50	55	20.5	
	51 and above	30	11.2	
Education	Junior High School	24	9.0	268
	Senior (Vocational) High School	36	13.4	
	University (College)	155	57.8	
	Graduate School	53	19.8	
Marital status	Unmarried (Including Single)	162	60.4	268
	Married	106	39.6	
Seniority	3 years or less	22	8.2	268
	3–5 years	97	36.2	
	5–8 years	75	28.0	
	8–11 years	33	12.3	
	More than 11 Years	41	15.3	

### Structural Equation Model Analysis

SEM analysis includes fit analysis and overall explanatory power of the research model. Scholars typically refer to seven numerical indices used for testing the overall model fit, including chi-square (χ^2^) test, χ^2^-degree of freedom ratio, goodness of fit index (GFI), adjusted goodness of fit index (AGFI), root-mean-square error (RMSEA), comparative fit index (CFI), comparative null model, and chi-square difference of independent model. The overall analysis results are presented in Table 2.

**Table 2:** Model fit analysis.

**Fit indices**	**Acceptable limit**	**This research model**	**Model fit judgment**
χ^2^ (Chi-square)	The smaller the better	22.183	
χ^2^-degree of freedom ratio	<3	1.63	fit
GFI	>0.9	0.98	fit
AGFI	>0.8	0.87	fit
RMSEA	<0.08	0.03	fit
CFI	>0.9	0.95	fit
NFI	>0.9	0.93	fit

*GFI, goodness of fit index; AGFI, adjusted goodness of fit index; RMSEA, root-mean-square error; CFI, comparative fit index; NFI, normed fit index*.

When testing model fit, a smaller χ^2^-degree of freedom ratio indicates better model fit. This research model showed a χ^2^-degree of freedom ratio <3 (1.63). GFI and AGFI indicate better fit when close to 1 and have no absolute standard to judge the fit although GFI > 0.9 and AGFI > 0.8 are acceptable. GFI and AGFI were 0.98 and 0.87, respectively, in this research. A good fit should show RMSEA less than 0.08—RMSEA was 0.03 in this study. The acceptable limit of CFI is >0.9—CFI in this study was 0.95. The normed fit index (NFI) should exceed 0.9—NFI in this study was 0.93. Overall, the goodness-of-fit indices conformed to the standards, revealing that the research results are acceptable. The sample data therefore could be used to explain the observation data.

From previous overall model fit indices, the model in this study presented favorable goodness-of-fit with observation data, showing that the theoretical model could fully explain the observation data. In this case, the correlation coefficients between synchronous online learning and learning motivation, learning satisfaction and the coefficient estimates could be further understood after the model fit test.

The research data are summarized in Table 3. The complete model analysis results reveal that five dimensions of synchronous online learning (role, participation method, participation location, interactive mode, and delivery method) could significantly explain synchronous online learning (*t* > 1.96, *p* <0.05), two dimensions of learning motivation (usefulness, enjoyment) could notably explain learning motivation (*t* > 1.96, *p* <0.05), and three dimensions of learning satisfaction (course content, teaching method, learning method) could effectively explain learning satisfaction (*t* > 1.96, *p* <0.05). Thus, the overall model in this study presents good preliminary fit.

**Table 3:** Overall linear structural model analysis result.

**Evaluation item**	**Parameter/evaluation standard**		**Result**
Preliminary fit	Synchronous online learning	Role	0.67^*^
		Participation method	0.70^*^
		Participation location	0.69^*^
		Interactive mode	0.71^*^
		Delivery method	0.73^**^
	Learning motivation	Usefulness	0.74^**^
		Enjoyment	0.72^**^
	Learning satisfaction	Course content	0.77^**^
		Teaching method	0.75^**^
		Learning method	0.79^**^
Internal fit	Synchronous online learning → learning motivation	0.88^***^
	Learning motivation → learning satisfaction	0.83^***^
	Synchronous online learning → learning satisfaction	0.85^***^

^*^p <0.05,

^**^p <0.01,

^***^p <0.001.

Regarding internal fit, synchronous online learning showed positive and significant correlations with learning motivation (0.88, *p* <0.01). Learning motivation revealed positive and significant correlations with learning satisfaction (0.83, *p* <0.01), and synchronous online learning appeared positively and significantly correlated with learning satisfaction (0.85, *p* <0.01). Thus, H1, H2, and H3 are supported.

The results show positive and significant correlations between synchronous online learning motivation, aligned with the research of Ettinger et al. (2006), Gresty et al. (2007), and Cheng et al. (2013). Learning motivation also revealed significant positive correlations with learning satisfaction, corresponding to the research of Barron (2006), Morgan et al. (2006), and Kohn et al. (2008). Finally, synchronous online learning was positively correlated with learning satisfaction, conforming to the research of Freed and Dawson (2006), Hayes and Dearnley (2007), and Chen et al. (2008).

## Discussion

The enhanced network transmission quality and the continuous development of software and hardware meet the needs for synchronous on-the-job online training. However, the promotion of e-learning in domestic on-the-job training still requires asynchronous e-learning. This approach lacks learning attraction for in-service learners. To promote synchronous online learning, information needs to be provided to public sector staff. For effective synchronous on-the-job online training, public sectors should familiarize staff with the Internet platform interface and make them aware of possible problems they might encounter so that staff can gain maximal learning effectiveness. The synchronous online learning instructors could record the operation process before the courses and establish video files on the network platform for staff to review online any time to enhance their computer self-efficacy. Instructors could also guide individual staff to increase familiarity with the synchronous online learning environment and establish successful computer use two or three times before the course. The video files of such courses could be used to demonstrate the training for new staff. After creating an environment with the appropriate equipment, staff with stronger computer competence could be encouraged to integrate computer use into the work environment. Such computer use would then serve as a benchmark in the working environment and could induce a learning atmosphere. A strategic decision of this nature could efficiently assist a team by using the right personnel to undertake activities that maximize effectiveness (Rodríguez and Böhme, 2009).

The development of future technology might exceed our imaginations. Learning styles might change significantly, and teachers may no longer lead learning. Synchronous online learning in the public sector should therefore keep up with social trends (Cheng et al., 2011), with teaching content planned in a careful and relevant way. The curriculum design should be centered on personnel working in the public sector using technology and media to aid in their development, create an optimal learning environment, and reinforce learning effectiveness (Yeh et al., 2007).

## Conclusion

Results revealed that public sector workers are motivated to participate in synchronous online learning, possibly because most staff cannot leave their work to participate in traditional on-the-job training. Synchronous e-learning is a convenient approach leading to greater motivation to participate in learning. As synchronous online learning can address the limits of traditional on-the-job training and tends to fit with the needs of staff, staff in the public sector show good learning satisfaction with online learning. These positive attitudes toward synchronous online learning mean that when difficulties are encountered they can be dealt with because of the belief in the ability to continue and make a success of their learning. Although staff might need to make considerable efforts, they typically do their best to achieve the goal and experience higher satisfaction.

Synchronous on-the-job online training is a new learning model for staff in the public sector and public sectors should establish comprehensive supporting measures to help employees fit the learning approach into their working day. Disciplinary actions and admonishment should be replaced with encouragement and support to eliminate a fear of the impact of computer technology and to further enhance the willingness to use computers. Previous research has often focused on online learning in schools but has rarely examined online learning in the public sector. The study discusses the effects of synchronous online learning on learning motivation and learning satisfaction in relation to public sector on-the-job training. We focused on actual learning conditions to reflect the real situation for public sectors that adopt or practice synchronous online learning in the future. The objective of this study was to determine the optimal learning program for on-the-job training in the public sector. The study contributes direction to researchers working in this area. Furthermore, this study can serve as a reference for academic research or practice. The Internet facilitates links between people and is becoming a mainstream medium for teaching practice. The practice of online learning can bring more benefits to more people. The research results contribute to the evaluation of the practicality of synchronous distance education for students in remote areas, different schools, and countries. The research helps to assess the benefits of the Ministry of Education's promotion of lifelong distance education and the distance education of general corporate employees. Synchronous online learning may be less suitable for the education and training of corporate employees, who might prefer self-study in their free time to make the learning more flexible.

## Data Availability Statement

The original contributions presented in the study are included in the article/supplementary material, further inquiries can be directed to the corresponding author/s.

## Ethics Statement

This study was reviewed and approved by the Ethics Committee of the Shih Hsin University. Written informed consent was obtained from all participants for their participation in this study.

## Author Contributions

NC performed the initial analyses and wrote the manuscript. I-JY assisted in the data collection and data analysis. All authors revised and approved the submitted version of the manuscript.

## Conflict of Interest

The authors declare that the research was conducted in the absence of any commercial or financial relationships that could be construed as a potential conflict of interest.

## Publisher's Note

All claims expressed in this article are solely those of the authors and do not necessarily represent those of their affiliated organizations, or those of the publisher, the editors and the reviewers. Any product that may be evaluated in this article, or claim that may be made by its manufacturer, is not guaranteed or endorsed by the publisher.
